# From Free Tissue Transfer to Hydrogels: A Brief Review of the Application of the Periosteum in Bone Regeneration

**DOI:** 10.3390/gels9090768

**Published:** 2023-09-21

**Authors:** Hai Xin, Eva Tomaskovic-Crook, D S Abdullah Al Maruf, Kai Cheng, James Wykes, Timothy G. H. Manzie, Steven G. Wise, Jeremy M. Crook, Jonathan R. Clark

**Affiliations:** 1 Integrated Prosthetics and Reconstruction, Department of Head and Neck Surgery, Chris O’Brien Lifehouse, Camperdown, NSW 2050, Australia; maruf.almaruf@lh.org.au (D.S.A.A.M.); kai.cheng@health.nsw.gov.au (K.C.); james.wykes@lh.org.au (J.W.); tim.manzie@lh.org.au (T.G.H.M.); jonathan.clark@lh.org.au (J.R.C.); 2 Central Clinical School, Faculty of Medicine and Health, The University of Sydney, Camperdown, NSW 2050, Australia; 3 Arto Hardy Family Biomedical Innovation Hub, Chris O’Brien Lifehouse, Camperdown, NSW 2050, Australia; eva.tomaskoviccrook@lh.org.au (E.T.-C.); jeremy.crook@lh.org.au (J.M.C.); 4 School of Medical Sciences, Faculty of Medicine and Health, The University of Sydney, Camperdown, NSW 2006, Australia; steven.wise@sydney.edu.au; 5 Intelligent Polymer Research Institute, AIIM Facility, Innovation Campus, University of Wollongong, North Wollongong, NSW 2500, Australia; 6 Royal Prince Alfred Institute of Academic Surgery, Royal Prince Alfred Hospital, Sydney Local Health District, Camperdown, NSW 2050, Australia

**Keywords:** periosteum, ossification, hydrogels, cambium layer, progenitor cells, synthetic periosteum, 3-D printing

## Abstract

The periosteum is a thin layer of connective tissue covering bone. It is an essential component for bone development and fracture healing. There has been considerable research exploring the application of the periosteum in bone regeneration since the 19th century. An increasing number of studies are focusing on periosteal progenitor cells found within the periosteum and the use of hydrogels as scaffold materials for periosteum engineering and guided bone development. Here, we provide an overview of the research investigating the use of the periosteum for bone repair, with consideration given to the anatomy and function of the periosteum, the importance of the cambium layer, the culture of periosteal progenitor cells, periosteum-induced ossification, periosteal perfusion, periosteum engineering, scaffold vascularization, and hydrogel-based synthetic periostea.

## 1. Introduction 

Bone tissue engineering (BTE) is dedicated to regenerating skeletal tissues for repairing both the form and function of damaged bones. It utilizes stem cell technology, biomaterials, and specialized fabrication techniques to initiate new bone growth. An important goal of BTE is to replace the current gold standard used in clinical practice, i.e., the use of autologous bone, which is often procedurally complex and time-consuming and leads to substantial donor site morbidity [1,2]. The design and manufacture of scaffolds are critical steps in tissue engineering as the scaffold provides a three-dimensional (3D) matrix for cell attachment and structural support for tissue development. For bone engineering, a scaffold should be amenable to osteoinduction, integrate with the host bone [2,3,4], and support tissue vascularization. Vasculature is particularly important for oncological maxillomandibular bone regeneration since the oral cavity harbors a diverse range of microorganisms and patients frequently undergo radiotherapy as part of cancer treatment, which inhibits the formation of new tissue. The inclusion of functional vasculature is crucial for large-size grafts and allows for the recruitment of highly specialized cells, such as tissue progenitors or immune cells, both of which contribute to bone regeneration and remodeling, with the latter also serving as a first line of defense against infection [5,6].

The periosteum is widely used in clinical practice to assist with the repair of bone defects [7]. In most instances, this is in the context of vascularized bone flaps in which the blood vessels are attached to the bone via the periosteum. However, clinical examples of the use of periostea to generate new bone have been reported. For instance, vascularized periosteal flaps taken from the iliac crest have been used in traumatic maxillary defects [8], and the combination of pericrania and temporoparietal fascial flaps has been used to heal mandibular defects caused by osteoradionecrosis [9]. In another clinical case, a periosteal sleeve was circumferentially elevated off a healthy diaphyseal bone adjacent to a bone defect following tumor resection. The healthy bone was then osteotomized and moved along an intramedullary nail to fill the gap, and osteogenesis was observed inside the periosteal sleeve that was left behind [10]. Spontaneous bone regeneration has also been reported from the periostea of vascularized fibula flaps used for mandibular repair [11]. 

As a thin layer of connective tissue covering the bone surface, the periosteum is a major source of osteogenic progenitor cells and blood supply to the bone. It is an essential component for bone development, facture healing, and regeneration [12,13]. Research on the use of the periosteum in BTE has expanded from simply transplanting the periosteum for in vivo bone growth to in vitro periosteal cell isolation and expansion [14], periosteal perfusion [15], and periosteum engineering [16]. Much of this research has involved hydrogels as promising materials for fabricating artificial periostea owing to their biocompatibility and tunable mechanical, osteoconductive, and osteoinductive properties [1,17]. Although many publications describe the different applications of the periosteum, there are no reviews that summarize the emerging research field of **periosteum enhanced bone tissue engineering (PEBTE)**. Thus, this paper aims to provide an overview of the research investigating periostea derived for bone repair, with consideration given to the anatomy and function of the periosteum, including the periosteal structure, the importance of the cambium layer, the culture of periosteal progenitor cells, periosteum-induced ossification, periosteal perfusion, periosteum engineering, scaffold vascularization, and hydrogel-based synthetic periostea. Hydrogels, as promising biomaterials for the fabrication of scaffolds, will be highlighted for their potential role in making a synthetic periosteum. 

## 2. The Histology of the Periosteum 

The periosteum is a thin layer of connective tissue that covers bone. It is composed of two sub-layers: an outer fibrous layer and an inner cambium layer. The outer fibrous layer has low cellularity but abundant nerves and blood vessels. This layer can be further divided into superficial and deep layers. The superficial layer contains few fibroblasts and therefore lacks elasticity but is highly vascularized and is the major contributor of the supply of blood to the underlying bones and attached skeletal muscles [12]. The deep layer exhibits a much higher degree of mechanical elasticity due to the large number of fibroblasts and elastic fibers but is relatively avascular [18]. In addition to supplying blood and nerves, the main role of the outer fibrous layer is to act as a pressure and tension buffer that stabilizes the hematoma during fracture healing [18,19,20]. 

One important characteristic of the periosteum is its mechanical anisotropy. This is likely due to the aligned orientation of collagen and elastin fibers which renders the periosteum pre-stressed. When a periosteum is procured from its underlying bones, it shrinks, with a larger degree of shrinkage in the axial direction along the bone than circumferentially [21]. Tensile tests conducted on periosteal flaps collected from sheep femora demonstrated that the elastic modulus of the axial specimens was five times larger than that of the circumferential specimens [21]. 

**Figure 1:**
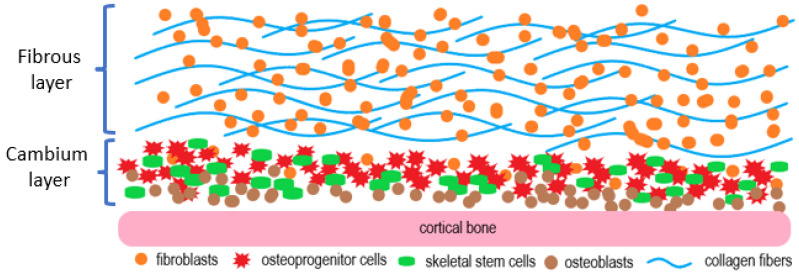
The periosteum contains an outer fibrous layer and an inner cambium layer. The cambium layer is the reservoir of osteochondrogenic progenitor cells, skeletal stem cells, osteoblasts, and others, while the fibrous layer is abundant in fibroblasts and collagen fibers.

The inner cambium layer is adjacent to the bone surface. It harbors various types of osteogenic cells including mesenchymal progenitor cells, differentiated osteogenic progenitor cells, osteoblasts, and pericytes distributed within a collagen matrix that are recruited to a fracture site for bone healing [19]. It should be noted that the thickness and osteogenic potential of the cambium layer declines with age but may be re-activated via mechanical stimulation [18,22]. The periosteum was shown to increase its cambium thickness and cellularity following surgical trauma in which incisions were made through the fibrous layer down to the cortical surface [23]. Apart from age, location is another critical parameter to consider in periosteal procurement [24]. Moore et al. studied the layer thickness and cambium cellularity of periostea collected from the tibias and femurs of deceased patients aged between 68 and 99 years. The thickness of the periosteum was approximately 100 µm overall, and the cambium layer from the tibia was 29 ± 3.1 µm compared to 23 ± 2.5 µm for the cambium layer from the femur [25]. For a given individual, the thickness and cellularity of the cambium layer on the tibia along the major centroidal axes is much higher than the respective layer on the femur. The results may provide a useful reference when harvesting periostea for bone reconstruction [25]. Figure 1 below gives a simplified demonstration of the structure and major cell types of the periosteum. 

## 3. Early, Contentious Studies of the Periosteum

The importance of the periosteum in bone regeneration was only recently broadly accepted, having been intensely debated during the 19th and early 20th centuries. Conflicting research involved various animal species (rabbits, rats, chicken, dogs, etc.), transplantation types, surgical procedures, and sources of periostea [26,27]. 

The earliest research dates back to the 18th century, with Duhamel placing a number of silver wires under the periostea of long bones and observing bone growth after several weeks [13,28]. Ollier, a French scientist, pioneered research investigating the osteogenic effect of the periosteum. Between 1859 and 1867, he performed a series of experiments demonstrating that multiple factors influence osteogenesis, including animal age, type, surgical technique, vascularity, the osteogenic stimulation of transplants, transplant size and location, immediacy, infection, and the integrity of the periosteal cambium layer [26,28]. Following Ollier’s work, attempts to test the potency of free periosteal transplants for ossification have resulted in both positive and negative results [26]. 

An important advance was made by Riess (1924), who found a close association between ossification and the presence of the cambium layer and its age. Periosteal transplants collected from young dogs tended to exhibit intact cambium layers and consistently supported new bone growth. However, no ossification was observed for periostea harvested from elderly dogs which had no cambium layer or fragmented cambium layers [26]. Riess’s conclusion echoed the viewpoint of Kolodny (1923), who emphasized that many studies did not differentiate between a young periosteum (at a reactive stage) and an old periosteum (at latent stage) and that with appropriate stimulation, periostea procured from adult animals should display an osteogenic potency equivalent to periostea collected from young animals [29]. In 1930, Burman and Umansky transplanted free periosteal grafts harvested from the tibias of young rabbits into the tendons of the tibialis anterior muscles. The research demonstrated new bone growth in the presence of the cambium layer and the opposite when the cambium layer was absent [26]. Schepelmann further studied the effect of vascularization, comparing periostea transplanted into either highly vascular organs (the liver, spleen, ovaries, etc.) or less vascular organs (the stomach or bowel). Better ossification was observed in the former group, showcasing the importance of blood supply [26]. To clarify the periosteum’s role in ossification, in his 1952 paper, Urist suggests that “the nineteenth-century controversy about the osteogenic potency of periosteum arose because it was not generally recognized that the negative results occurred only in transplants from adult or old animals” [30]. Based on the above-mentioned studies, we note that there were several key factors determining the ossification efficacy of the periosteum. Among them, age, vascularization, and the integrity of the periosteal cambium layer should be highlighted and carefully considered for experimental planning. 

## 4. Periosteum-Induced Intramembranous and Endochondral Ossification

Bone fracture healing can be categorized into two groups: stabilized fracture healing and non-stabilized fracture healing [19,31]. In the former group, periosteal cells are minimally stimulated. If there is no gap between two fracture ends, a repair process called contact healing will take place wherein oriented lamellar bone is produced by osteoprogenitor cells derived from the Haversian system within the cortex. When the fracture gap is small, lamellar bone forms first and is subsequently remodeled to the correct orientation. When the fracture gap is wide, woven bone is generated, followed by its conversion into lamellar bone [18]. 

**Figure 2:**
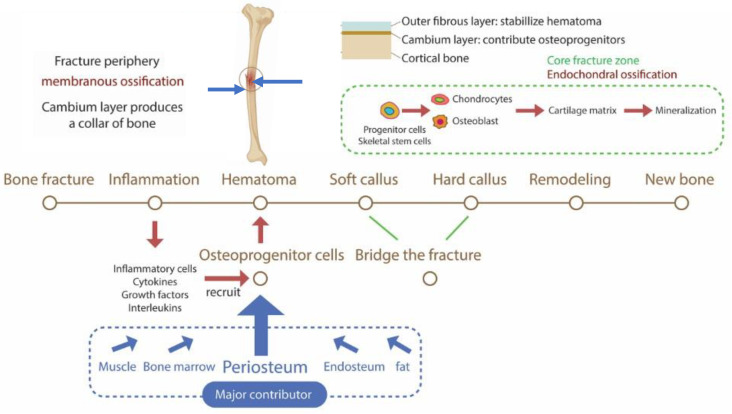
The periosteum is the major contributor to intramembranous and endochondral ossification for bone fracture healing.

In non-stabilized fracture healing, periosteal cells respond to mechanical stimuli to induce bone healing through several stages [18]. Following bone fracture, the hematoma plays a significant role in bone repair by not only providing a “recovery medium” but also by releasing cytokines to recruit osteogenic stem cells from various sources, including the periosteum, bone marrow, and endosteum [20]. In the peripheral zones of the fracture where vascularization is maximal, the outer fibrous layer of the periosteum acts as a mechanical stabilizer, and intramembranous ossification occurs via the differentiation of mesenchymal stem cells derived from the periosteum into osteoblasts. The differentiated osteoblasts secrete osteoid, which calcifies to form bone tissue [19,31]. However, in the center of the fracture site, where the vascularization is poor, progenitor cells from the cambium layer of the periosteum aggregate in the hematoma and differentiate into chondrocytes that subsequently form cartilage (soft callus) to bridge the fracture. These cartilaginous tissues then mineralize (hard callus) to form bone. This repair mechanism is called endochondral ossification [18,19,31]. Therefore, the periosteum plays a major role in fracture healing by providing osteochondrogenic progenitor cells. A fracture repaired via endochondral ossification differs from intramembranous ossification in that it involves an intermediate stage in which cartilage is formed and then ossified [12]. Figure 2 demonstrates the role played by the periosteum in bone fracture repair. 

## 5. The Periosteum Contains Skeletal Stem Cells That Undergo Chondrogenic and Osteogenic Differentiation in Response to BMP-2 

As the cambium layer of the periosteum provides critical osteochondrogenic cells that contribute to both endochondral and intramembranous ossification, the use of these skeletal stem cells for BTE requires their characterization. Another important question is whether periosteal cells outperform other stem cell sources such as bone marrow and endosteum in terms of bone regeneration. Researchers have isolated both bone marrow stromal/skeletal stem cells (BMSCs) and periosteal cells from mouse tibias and femurs [14]. They demonstrated that BMSCs and periosteal cells share a common mesenchymal embryonic origin, but periosteal cells exhibit a better bone regenerative capacity [14]. Although BMSCs are important in regulating hematopoiesis and bone resorption, they play indirect roles in skeletal repair by releasing growth factors. On the other hand, periosteal cells exhibit the critical characteristics of skeletal stem cells by expressing genes that show stemness and skeletal system development and are thus major contributors to the formation of cartilage and bone formation calluses for skeletal repair [14]. The research team also identified a crucial extracellular matrix protein called Periostin as a key regulator of skeletal stem cells in the periosteum. Compared to wild-type periosteum, Periostin-depleted mice have shown impaired bone repair and an inability to reconstitute a pool of periosteal cells in response to the injury [14,32]. 

In other work, segmental mandibular defects were created in 18-month-old mini-pigs and fixated with a reconstruction plate. The periosteum was then sutured back, thus recreating the periosteal envelope. The periosteal tissues from the experimental and control sites were collected in week 1 and week 2, respectively. A histological assessment of the cambium layer found more tissue growth in week 2 than that in week 1, with the over-expression of several osteogenesis-associated genes involved in Tgfβ/Bmp, Wnt, and Notch signaling pathways [33]. Bone morphogenetic protein (BMP)-2 was suggested to be the key regulator of periosteum-induced bone regeneration [33,34]. As reported in several other publications, BMP-2 knockout mice exhibited a number of negative consequences for bone repair, including delayed periosteal activation [35], the absence of a bridging callus [35], un-differentiated progenitors [36,37], and prolonged cartilage callus growth [37]. Thus, BMP-2 is necessary for periosteum-induced bone healing. 

Recombinant human (rh)BMP-2 preferentially targets the periosteum, which expresses BMP-2 receptors in the early stages of bone repair to activate the BMP signaling pathway. rhBMP-2 recruits skeletal progenitor cells from both the periosteum and endosteum and promotes both the chondrogenic and osteogenic differentiation of skeletal progenitor cells from the periosteum but only the osteogenic differentiation of endosteal progenitor cells. This highlights a key difference between periosteal and endosteal cells, i.e., the response to BMP-2 stimulation [34]. 

## 6. The Procurement of the Cambium Layer

The importance of maintaining the cambium layer for periosteum-induced osteogenesis has been recognized as early as the 19th century. However, detaching an intact cambium from the underlying bone is a challenging task. Firstly, the cambium layer is only ~20 µm or from two to three cells thick and decreases with age [25]. Secondly, the cambium layer is attached to cortical bone, sending a great many “buds” into the superficial pores of the bone [38]. Once the layer is elevated, the buds containing osteogenic progenitor cells may still remain on the bone, which greatly reduces the osteogenic efficacy of the harvested periosteum [38]. A third important factor is the elevation technique. Brownlow attempted to harvest humeral and tibial periostea from adult white rabbits via four different techniques including pulling, sharp dissection, the use of a periosteal elevator, and chisel elevation. They recommend using either a periosteal elevator or chisel as both tools are able to strip both fibrous and cambium layers, even though chisel elevation may create significant surface damage to the underlying bone. In contrast, using a scalpel or pulling technique left the cambium layer attached to the bone surface [39]. From other work, clinicians procured periosteal samples at 1 cm, 3 cm, and 5 cm proximal and distal to the fracture sites of long bones in 20 patients. However, they found the majority of the harvested periostea only contained the outer fibrous layer, while the inner cambium layers remained attached to the bone surfaces [40]. Simon et al. stimulated the proliferation of the cambium layer by incising the periosteum and scoring to the cortical bone. The wound was then closed to allow for in situ periosteal proliferation for 4, 8, and 16 days, respectively. Following animal euthanasia, the periostea were harvested, and the results demonstrate that the expression of BMP-2 was significantly elevated in the harvested periostea [23]. 

## 7. Periosteal Cell Isolation, Expansion, and Characterization

The osteogenic progenitor cells contained in the cambium layer of the periosteum can be isolated and expanded in vitro. There are two approaches used for this purpose: explant culture and tissue digestion [41]. The former approach involves culturing harvested periosteal tissues wherein the periosteal cells migrate and outgrow from the primary tissue. The tissue digestion method utilizes various collagenases to isolate the cells from a digested collagen matrix [13,41]. It is worth noting that the osteogenic and chondrogenic capacity of a harvested periosteum decreases within hours. Thus, delays in the harvesting procedure need to be avoided and once harvested, the explants should be placed in Ringer’s solution, phosphate-buffered saline, or a serum-free culture medium within 10–15 min [13]. 

One of the early attempts to culture periosteal explants employed 2-month-old New Zealand white rabbits to investigate the effects of time and storage temperature on the chondrogenic potential of the periosteal explants. The periosteal tissues were harvested from cadavers stored at room temperature or 4 °C for 0, 4, 6, 8, 12, 16, 18, or 24 h following sacrifice. The explants were cultured for 6 weeks using an organ culture model [42], and a standard cartilage yield assay was performed [43]. The periosteal explants stored at room temperature for ≥4 h showed little chondrogenesis, while chondrogenesis was observed if the deceased animals were stored at 4 °C for no more than 24 h. It is clear that the chondrogenic capacity of periosteal explants decreases quickly post-mortem or shortly after harvest [43]. A more recent study using the explant culture method flushed the bone marrow of mouse tibias and femurs, and the bone explants were cultured in growth media to obtain periosteal cells for characterization [14]. 

The enzymatic digestion method uses collagenase to decompose the collagen matrix and release the periosteal cells. The literature has reported the applications of collagenase-1 [44], -2 [45,46,47,48], -4 [49,50,51], and -6 [52], and an unspecified collagenase [53,54,55,56,57,58,59] on different sources of periostea, including the tibia [45,55], femur [56], mandible [44,52], and rib [59]. The harvested periosteum is minced finely and then digested with the enzyme for a length of time between 1 h [54] and overnight [57]. The widely used digestion media are Dulbecco’s Modified Eagle Medium (DMEM) [55,59], high-glucose DMEM [47,49,50], modified BGJb media [57], and Hank’s balanced salt solution [48]. Base media are usually supplemented with 10% fetal bovine serum and penicillin–streptomycin. Following the centrifugation of the digestion media, the collected cell pellets are re-suspended in the growth media for various research purposes [41]. 

Another critical task is to characterize the “stemness” of these isolated cells. Skeletal stem cells derived from the periosteum, bone marrow, and endosteum share a common mesenchymal origin. Mesenchymal stem cells (MSCs) are multipotent stromal cells which can self-renew and differentiate into the lineages for cartilage, bone, adipose, and skeletal muscle [54,60]. Given that there is still a lack of established criteria to identify periosteum-derived progenitor cells, the classic criteria used to identify MSCs can also be used to verify periosteum-derived stem cells [61]. According to the International Society for Cellular Therapy, the minimal criteria for the identification of MSCs is the expression of surface markers of the clusters of differentiation CD73, CD90, and CD105 and a lack of expression of CD45, CD14, CD34, and HLA-DR (Human Leukocyte Antigen-DR isotype) [61]. To differentiate the pure periosteum-derived progenitor population from fibroblasts, one should also consider other markers. For example, MSCs express more CD166 than fibroblasts do, whereas the levels of expression of CD9 are higher in fibroblasts than in MSCs [62]. In a study using a real-time polymerase chain reaction (PCR), the researchers found that MSCs exhibited levels of expression of CD106, integrin alpha 11, and insulin-like growth factor-2 that were 10-fold higher than fibroblasts, while the expression levels of matrix metalloproteinase 1 and matrix metalloproteinase 3 in the MSCs were 100-fold lower than in fibroblasts [62]. Some other markers which may be useful for the identification of periosteum-derived progenitor cells include Leptin receptor [63,64], Prrx1 [65,66], Periostin [14], and Nestin [67,68]. 

## 8. In Vivo and Ex Vivo Periosteal Bioreactor Systems

In addition to using periosteum-isolated cells, another approach is to incorporate a vascularized periosteum flap into a scaffold that can be transplanted for bone regeneration. A periosteum was used to enclose in vivo bioreactors containing bone scaffolds to stimulate osteogenesis within and vascularize the scaffold [69,70]. Tatara et al. used a rib periosteum to generate large amounts of mineralized bone that were transplanted for mandible reconstruction [71]. Huang et al. employed a vascularized periosteal flap to revascularize a decellularized bone matrix scaffold in a rabbit model [72]. In a sheep model, a vascularized periosteum was elevated from the infraspinous scapula and wrapped around an autologous bone graft. The periosteum-wrapped bone grafts were incubated in the sheep’s body for 84 days. The results demonstrated a thickening of the periosteal flap with endochondral ossification [73]. 

**Figure 3:**
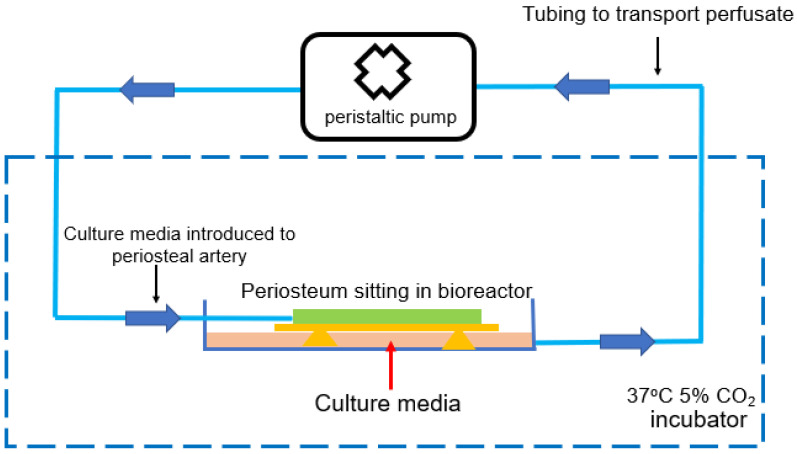
The concept of ex vivo perfusion bioreactor system to maintain the tissue viability of a periosteum procured from an ovine model, adapted from [15]. In the system, the culture media containing oxygen and nutrients were introduced into the periosteal vascular network via its artery, using a circulating pump. The cellular viability of the tissue was reported to be preserved for up to 4 weeks [15].

However, the use of a periosteum for scaffold fabrication requires a feasible ex vivo approach in which the tissue viability of the harvested periosteum is preserved. Xin et al. used a perfusion bioreactor system as demonstrated in Figure 3 in which an ovine periosteal flap was placed in a 3-D printed bioreactor and perfused with a culture medium via its artery [15]. Both live and dead assays, PrestoBlue assays, and a histology analysis suggested that a significant proportion of cells were still viable after being perfused for up to 4 weeks. As a proof-of-concept study, the proposed ex vivo perfusion system was shown to be a feasible solution for preserving a harvested periosteum for a prolonged period for upcoming scaffold vascularization. However, several key system parameters still need to be optimized, such as the flow rate, pressure, and culture medium formulation [15]. These factors have been proven important in previous similar studies in which the researchers attempted to perfuse scaffold constructs seeded with osteogenic cells to study bone biology, proliferation, and gene expression [74,75,76]. Meanwhile, maintaining an efficient oxygen supply to the perfused tissue over the course of an experiment is another critical issue [77].

## 9. Hydrogel-Based Artificial Periostea for Bone Regeneration

The utilization of harvested periostea is limited by several factors such as availability, tissue viability, tissue morphology, age, and the morbidity of procurement. Periosteum engineering or artificial periostea has the potential to provide customizable and biomimetic periostea bespoke for different clinical applications. Cell sheet techniques have been developed to form synthetic periostea in which the target cells are extracted, cultured, expanded, and fused, and an extracellular matrix is then produced to form a continuous and adhesive tissue sheet [19,78,79]. One example is the application of cell sheets formed by culturing cells isolated from a human mandible periosteum. The resultant sheet, together with platelet-rich plasma and hydroxyapatite granules, was used for the treatment of bony periodontal defects [80]. Another study achieved the formation of new bone based on BMSC-laden tricalcium phosphate (TCP) scaffolds encased by a cell sheet produced from periosteum-derived cells [81]. 

Another approach to fabricating a synthetic periosteum is to utilize hydrogels to form either single- or multi-layered scaffold membranes in which the osteogenic cells and growth factors are encapsulated to mimic the structure and function of a physiological periosteum [16,19]. Hydrogels are 3D polymeric networks with excellent water-absorbing capabilities, biocompatibility, flexibility, injectability [82], stimuli-responsiveness (pH, temperature, magnetic field, etc.) [83,84,85], and tunable mechanical properties [86]. All these characteristics make hydrogels ideal material candidates for various biomedical applications including drug delivery [87,88], tissue engineering scaffolds [89,90], artificial muscles [91,92], wound healing [93], wearable sensors [94,95], cornea repair [96,97], and cartilage replacement [98,99]. Compared with synthetic polymers in which un-reacted crosslinkers or monomers may introduce cytotoxicity [100,101], biopolymers such as collagen, gelatin, chitosan, alginate, and Gelatin Methacryloyl (GelMA) exhibit enhanced biocompatibility and have broad applications as bioactive scaffolds in bone tissue engineering.

One of the most studied biopolymers is collagen, which represents a family of 28 subcategories of proteins with similar triple-helix structures composed of inter-twined polypeptide chains. Collagen type 1 is especially abundant and is a key component of bone, cartilage, cornea, tendons, and ligaments [102,103,104]. As an important constituent of the extracellular matrix (ECM), collagen type 1 is widely used as a scaffold material to provide mechanical support and biological cues for cell proliferation and differentiation [105,106]. For example, to develop alternatives to autologous bone grafts in posterolateral spinal fusion, mesenchymal stem cells (MSC) have been cultured in type 1 collagen gels with hydroxyapatite particles, resulting in a favorable degree of spinal fusion in adult rabbits [107]. More recently, a hydrothermal approach has been applied to prepare nanoscale, rod-like hydroxyapatite particles which were incorporated into type 1 collagen hydrogels to form a homogeneous composite to mimic bone structures. A particularly significant breakthrough was the application of Darvan 821-A as both a particle-size-controlling agent and dispersion agent to prevent the agglomeration of hydroxyapatite particles in collagen matrixes [108]. Apart from inorganic particles, collagen can be mixed with organic materials or other biopolymers to fabricate hydrogel scaffolds for osteogenesis and chondrogenesis. For example, chitosan was combined with bovine type 1 collagen, and the mixture was gelated with the cyto-compatible crosslinker glyoxal. The resultant hydrogel scaffold supported the attachment, proliferation, and osteogenic differentiation of human bone marrow stem cells [109]. Another example are collagen-alginate hydrogels, which inhibited the dedifferentiation of chondrocytes when compared with pure collagen hydrogels [110]. 

Another intensely researched biopolymer is GelMA, which has been shown to have the potential to repair segmental bone defects in rat models with bone marrow stem cells [111]. GelMA hydrogels are commonly prepared via UV light crosslinking, but the exposure of cells and tissues to UV light must be optimized to avoid DNA damage [112]. Visible light is a better alternative to activate the free radicals of riboflavin for GelMA crosslinking. GelMA-Riboflavin hydrogels seeded with KUSA-A1 cells were shown to exhibit elevated cellular viability, osteoblastic differentiation, and the expression of osteogenesis-associated genes when compared with UV-crosslinked GelMA hydrogels [113]. GelMA can also be combined with silver-containing hydroxyapatite microspheres to prepare injectable hydrogels. The hydrogel scaffold may be used to encapsulate a pre-osteoblast cell line (MC3T3-E1), resulting in enhanced mechanical toughness, cytocompatibility, and antimicrobial properties against *Staphylococcus aureus* and *Escherichia coli* [114]. In order to improve the mechanical properties and porosity of GelMA hydrogels, hydroxyapatite microtubules may be incorporated into GelMA matrixes to elevate the mechanical performance of the scaffold and to enhance the inter-connections of the hydrogel pores. These improvements serve to increase the cellular proliferation and differentiation of bone marrow mesenchymal stem cells [115]. Owing to its excellent printability, GelMA has been well utilized as a cell-laden bioprinting ink for tissue engineering [116], with recent studies supporting the addition of hydroxyapatite [117], silica nanoparticles [118], and synthetic polymers [119] to improve the mechanical properties and bioactivity of GelMA based bioinks. 

The use of hydrogels to prepare a synthetic, engineered periosteum was first proposed by Hoffman and Benoit in 2012; they indicated that allografts exhibited slow regeneration and minimal engraftment, whereas autograft healing was complete due to periosteum-mediated bone regeneration [120]. Moreover, they recommended using degradable poly (ethylene glycol)-poly (lactic acid)-dimethacrylate hydrogels as synthetic periostea encapsulating MSCs to fill and regenerate 5 mm segmental bone defects. Several advantages of using PEG for periosteum engineering included its resistance to nonspecific protein adsorption, tunable degradability, cellular encapsulation density, hierarchical network structures, and ability to release small-molecule drugs [120]. The same research group later described seeding MSCs into hydrolytically degradable PEG-based hydrogels and the encapsulation of the cells to the allograft surface. To achieve a 14-day survival of stem cells similar to what is observed during autograft healing, the degradable units within the hydrogel network were carefully altered through enhanced vascularization, bone callus formation, and increased biomechanical strength after 16 weeks of implantation. However, compared with autograft healing, the endochondral ossification of tissue engineered periosteum was found to be delayed [121]. To overcome this challenge and better mimic the native periosteal production of paracrine factors, including vascular endothelial growth factor (VEGF) and bone morphogenetic protein 2 (BMP2), a mixture of MSCs (50%) and osteoprogenitor cells (50%) was encapsulated within the tissue-engineered periosteum. Enhanced bone callus formation and graft–host integration were observed following in vivo implantation [122]. Other synthetic periostea have utilized GelMA. GelMA hydrogels can be blended with calcium phosphate nanoparticles to form an inorganic–organic hybrid construct which is then mixed with hydrogel fibers to mimic the periosteum. Human umbilical vascular endothelial cells and MC3T3-E1 cells were co-cultured into this artificial periosteum to stimulate both osteogenesis and angiogenesis [123]. 

For a better simulation of a natural periosteum, bi-layered or multiple-layered hydrogel membranes can also be considered. Alginate hydrogels can be crosslinked with varying amounts of hydroxyapatite nanoparticles to form two-membrane composites with one porous layer seeded with fibroblasts and one rougher and more mineralized layer growing with osteoblasts. Osteoblast differentiation was observed from the membrane with the highest concentration of hydroxyapatite [124]. Utilizing a layer-by-layer bottom-up strategy, researchers incorporated collagen with polycaprolactone (PCL) and nano-hydroxyapatite to prepare a synthetic composite which was seeded with BMSCs. This engineered periosteum was used along with a structural bone allograft to repair a segmental bone defect in a mouse femur [125]. 

**Figure 4:**
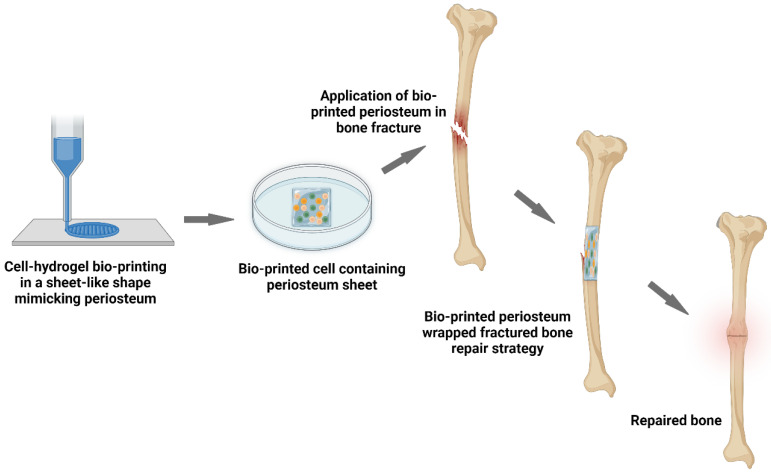
Three-dimensional bioprinting was used to prepare an engineered artificial periosteum for bone defect repair (Created with BioRender.com).

Additionally, 3-D printing techniques have demonstrated their utility for the production of customizable, precise, and hierarchical microstructures for scaffold constructs [4,126]. Various materials including degradable (iron, magnesium, and zinc) and non-degradable (titanium) metals [126], bioactive ceramics (hydroxyapatite [127]), non-degradable polymers (poly ether ether ketone [128]), degradable polymers (such as polycaprolactone [129] or poly lactic acid [130]), and hydrogels (gelatin and alginate [131] or GelMA [132]) can be printed into pre-designed and validated implantation constructs to repair bone defects. The degradable materials are those which can decompose via hydrolytic, enzymatic, cellular-mediated, or stimuli-assisted reactions in the body. The most crucial parameters when using a degradable scaffold for bone engineering are the degradation kinetics, which must be consistent with the growth of new bone [133,134]. By using 3-D printing techniques, Sun et al. manufactured a cambium layer based on GelMA mixed with nano-hydroxyapatite and a fibrous layer based on poly (N-acryloyl 2-lycine) (PACG) and GelMA loaded with Mg^2+^. The nano-hydroxyapatite in the biomimetic cambium layer was used to stimulate bone regeneration due to its favorable interaction with the host bones and its release of Ca^2+^. The hydrogen bonds formed by the PACG in the GelMA hydrogel layer not only enhanced the mechanical properties of the artificial fibrous layer but also extend its degradation time to 60 days. The addition of Mg^2+^ improved both angiogenesis and bone mineralization [132]. Figure 4 below demonstrates the concept of using 3-D bioprinting to manufacture a hydrogel-based, biomimetic, engineered periosteum as a bone regeneration scaffold.

Conventional hydrogels possess poor mechanical properties [135] and are unable to simulate the mechanical ductility and flexibility of a natural periosteum. This issue can be resolved through the use of several novel mechanically strong and tough hydrogels such as nano-composite hydrogels [136], slide-ring hydrogels [137], double-network hydrogels [138,139], covalent–ionic hybrid hydrogels [140,141], and hydrogen-bonded polyurethane hydrogels [142,143]. These tough hydrogels can be generally categorized into two groups: covalently crosslinked and physically crosslinked. Covalently crosslinked tough hydrogels, such as chemically crosslinked double-network hydrogels, possess a wide molecular-weight distribution in network strands, and these short network strands can be easily broken via small mechanical strains [144]. Physically crosslinked hydrogels possess reversible crosslinks, making the hydrogel network degradable and damage-recoverable, and are promising candidates as re-absorbable scaffolds to release and deliver osteogenic cells or molecules to treatment sites. 

A yet-unresolved challenge is the supply of nutrients to complex tissue-engineered constructs that are designed to be implanted in vivo to replace critical-sized bone defects. Whilst diffusion is feasible for thin artificial periostea, it is insufficient when combined with cellular bone scaffolds that are designed for hostile environments that have failed to repair via endogenous mechanisms. Therefore, the vascularization of artificial periostea needs to be addressed. One effective approach was to differentiate rat-bone-marrow-derived mesenchymal stem cells to induce endothelial-like cells. The obtained cells were combined with a cell sheet of un-differentiated MSCs. This sheet was used as an artificial fibrous layer. Meanwhile, another cell sheet was produced by differentiating the MSCs into osteogenic cells to form an artificial cambium layer. Both layers were assembled together to bio-mimic a natural periosteum, and the assembly was then wrapped around a porous β-tricalcium phosphate scaffold to facilitate vascularization [145]. In another approach, a decellularized periosteum was utilized as a template to create a pseudo-periosteum based on collagen in which the stem cells and endothelial cells were co-cultured to stimulate both osteogenesis and angiogenesis. The publication emphasizes the role of “cross-talk” between osteoblasts and endothelial cells for bone regeneration [146]. 

## 10. Summary and Future Directions

The periosteum plays essential roles in bone development and fracture healing. The human application of the periosteum for bone regeneration began a few centuries ago. Despite controversy, several scholars in the 19th and early 20th centuries emphasized the critical roles of the cambium layers, age, vascularization, and harvesting techniques. The periosteum contributes to both intramembranous and endochondral ossifications during bone fracture healing which, in turn, depends on stabilization and the distance to the fracture site. The cambium layer acts as a reservoir of osteogenic progenitor cells that are stimulated to differentiate by BMP-2 and skeletal stem cells, which have common mesenchymal origins with BMSCs. The cambium layer is thin, reduces with age, and is adherent to the cortical bone, making surgical harvesting challenging. The progenitor cells of the cambium cells can be isolated and expanded from a harvested periosteum via either explant culture or enzymatic digestion, and classical markers used for the identification of MSCs can also be used to characterize the cambium progenitor cells as distinct from periosteal fibroblasts. An ex vivo perfusion bioreactor system demonstrated the feasibility of preserving the cellular viability of a harvested periosteum. The preserved periosteum may have the potential for use in future scaffold fabrication and vascularization. Periosteum engineering or the creation of artificial periostea using hydrogel scaffolds is a growing and promising field. Osteogenic cells and molecules are encapsulated into single-layered or bi-layered hydrogel membrane scaffolds to mimic a natural periosteum. 3-D printing techniques can be applied to manufacture biomimetic artificial periostea with complex microstructures, with one key challenge being the vascularization of the constructs. In the future, ex vivo perfusion may be combined with artificial periostea to expand their application to complex bone scaffold constructs for hostile tissue beds. This research direction may present a new perspective to accelerate the development of bone tissue engineering. 

## Data Availability

Not applicable.
